# Effect of Environmental Pollutants PM2.5, CO, NO_2_, and O_3_ on the Incidence and Mortality of SARS-CoV-2 Infection in Five Regions of the USA

**DOI:** 10.3390/ijerph18157810

**Published:** 2021-07-23

**Authors:** Sultan Ayoub Meo, Abdulelah Adnan Abukhalaf, Omar Mohammed Alessa, Abdulrahman Saad Alarifi, Waqas Sami, David C. Klonoff

**Affiliations:** 1 Department of Physiology, College of Medicine, King Saud University, Riyadh 11461, Saudi Arabia; abdall-1234@hotmail.com (A.A.A.); omar.m.alessa@gmail.com (O.M.A.); Abdulrahman.s.a222@gmail.com (A.S.A.); 2 Department of Public Health, University of Health Sciences, Lahore 54600, Pakistan; biostatistician1@gmail.com; 3 Mills-Peninsula Medical Center San Mateo, Diabetes Research Institute, San Mateo, CA 99401, USA; dklonoff@diabetestechnology.org

**Keywords:** environmental pollution, COVID-19, prevalence, mortality, USA

## Abstract

In recent decades, environmental pollution has become a significant international public problem in developing and developed nations. Various regions of the USA are experiencing illnesses related to environmental pollution. This study aims to investigate the association of four environmental pollutants, including particulate matter (PM2.5), carbon monoxide (CO), Nitrogen dioxide (NO_2_), and Ozone (O_3_), with daily cases and deaths resulting from SARS-CoV-2 infection in five regions of the USA, Los Angeles, New Mexico, New York, Ohio, and Florida. The daily basis concentrations of PM2.5, CO, NO_2_, and O_3_ were documented from two metrological websites. Data were obtained from the date of the appearance of the first case of (SARS-CoV-2) in the five regions of the USA from 13 March to 31 December 2020. Regionally (Los Angeles, New Mexico, New York, Ohio, and Florida), the number of cases and deaths increased significantly along with increasing levels of PM2.5, CO, NO_2_ and O_3_ (*p* < 0.05), respectively. The Poisson regression results further depicted that, for each 1 unit increase in PM2.5, CO, NO_2_ and O_3_ levels, the number of SARS-CoV-2 infections significantly increased by 0.1%, 14.8%, 1.1%, and 0.1%, respectively; for each 1 unit increase in CO, NO_2_, and O_3_ levels, the number of deaths significantly increased by 4.2%, 3.4%, and 1.5%, respectively. These empirical estimates demonstrate an association between the environmental pollutants PM2.5, CO, NO_2_, and O_3_ and SARS-CoV-2 infections, showing that they contribute to the incidence of daily cases and daily deaths in the five different regions of the USA. These findings can inform health policy decisions about combatting the COVID-19 pandemic outbreak in these USA regions and internationally by supporting a reduction in environmental pollution.

## 1. Introduction

Over the past three decades, environmental pollution has become an increasingly serious global public health problem. Progressive urbanization after the industrial revolution has increased the amount of environmental pollution to dangerous levels. Various sources of pollution have changed the composition of the environment and climatic conditions [1,2]. Environmental pollution results from unfavorable changes caused by the direct or indirect action of human-associated activities [3]. The environment affects the physiology and psychology of individuals. The environment incorporates the biotic: “living organisms, and microorganisms”; and the abiotic: the “hydrosphere, lithosphere, and atmosphere” [4].

In recent years, the epidemiological literature has established that environmental pollution is associated with multiple adverse outcomes in humans, including acute and chronic respiratory infections, chronic obstructive pulmonary disease (COPD), asthma, coronary artery diseases, and lung cancer [4]. The literature also demonstrates that environmental pollution can increase the number of SARS-CoV-2 cases and deaths globally [1]. As of 5 April 2021, the total number of documented SARS-CoV-2 cases worldwide was 131,487,572, and the total number of deaths was 2,857,702 (2.17%). However, in the USA, the total number of cases of SARS-CoV-2 was 568,801,23, which comprised 43.25% of the total reported global SARS-CoV-2 cases [5].

Environmental pollutants, including particulate matter (PM2.5), carbon monoxide (CO), nitrogen oxides (NO_2_), ozone (O_3_), and volatile organic compounds (VOCs), are commonly found in high concentrations in large cities [6]. Environmental and weather conditions can impact health and disease [7,8,9]. Environmental pollution may promote the spread of microbes and SARS CoV-2 infection [10,11]. This study aimed to investigate the relationship of environmental pollutants PM2.5, CO, NO_2_, and O_3_ with the numbers of SARS-CoV-2-related daily new cases and daily deaths in five different regions of the USA: Los Angeles, New Mexico, New York, Ohio, and Florida.

## 2. Subjects and Methods

**Figure 1:**
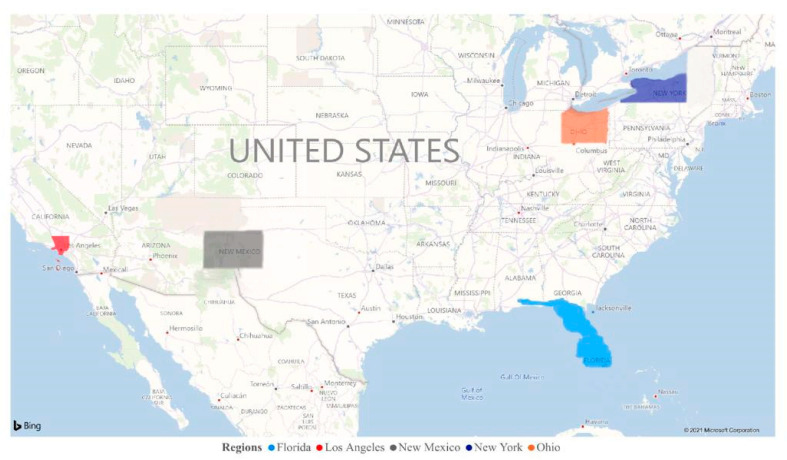
Map of the USA, highlighting five selected regions. Florida: light blue; Los Angeles: red; New Mexico: gray; New York: dark blue; and Ohio: orange.

The present research study analyzed the impact of four environmental pollutants, namely particulate matter PM2.5, CO, NO_2_, and O_3_, in five different regions of the USA (Figure 1) and their association with new cases of SARS-CoV-2 and deaths from COVID-19. In this study, we selected five different regions of the USA: Los Angeles, New Mexico, New York, Ohio, and Florida (Figure 1). We recorded the number of daily new cases and deaths and their possible association with environmental pollutants in these regions.

The SARS-CoV-2 data relating to the daily basis number of cases and deaths were recorded from Worldometer Web [12]. The daily basis concentrations of PM2.5, CO, NO_2_, and O_3_ were documented from two metrological websites, the United States Environmental Protection Agency-EPA [13] and the Real-Time Air Quality Index—AQI [14]. The data were obtained starting from the first case of (SARS-CoV-2) in any of these regions from 13 March to 31 December 2020.

### 2.1. Ethical Statement

The data samples were gathered at the regional level and did not directly involve patients or their medical records. As a result, no formal approval from an institutional ethical review board was required.

### 2.2. Statistical Analysis

The results were analyzed by R Core Team (2020) for Statistical Computing, Vienna, Austria. Microsoft Power BI. (desktop version) was used for some of the data visualizations, including maps. The data ordinariness for normal and Poisson distributions was carefully checked using a one-sample Kolmogorov–Smirnov test. The median (25th–75th) quartiles are presented for non-normally distributed quantitative variables. Spearman Rho correlation was applied to evaluate the association between various meteorological factors at a 1% level of significance, while Poisson Regression Analysis was used to envisage the number of cases and deaths from PM2.5, CO, NO_2_ and O_3_. The goodness of fit tests and model tests for regression analysis was significant at a 5% level of significance.

## 3. Results

**Figure 2:**
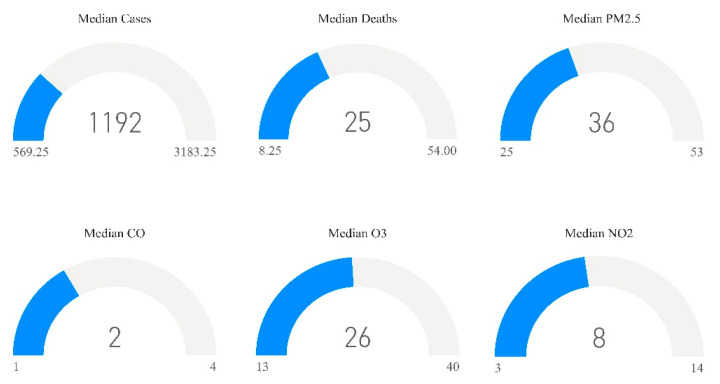
Median (25th–75th) quartiles of study parameters.

**Figure 3:**
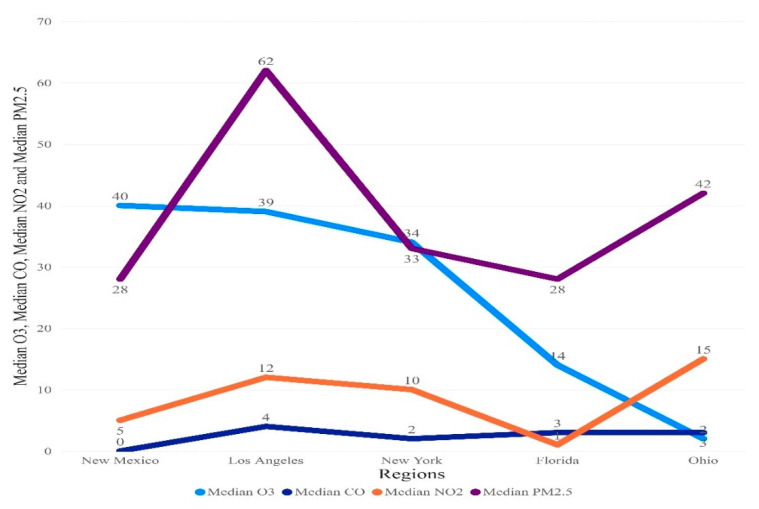
Median of pollutant parameter by region.

**Figure 4:**
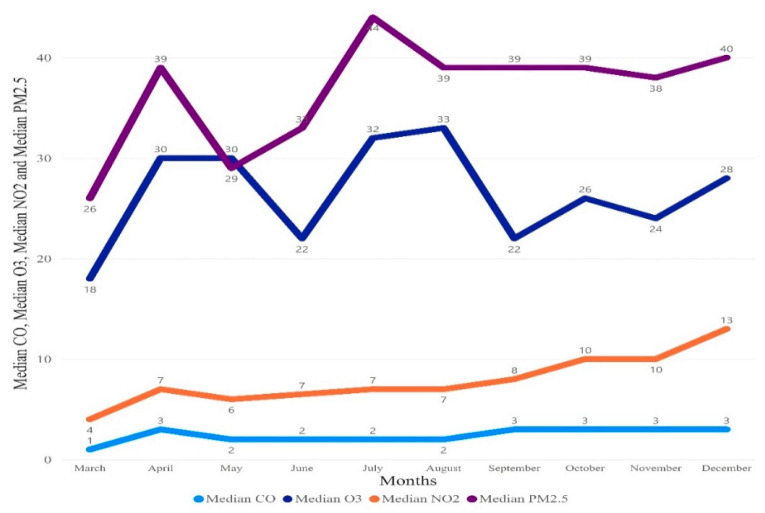
Monthly median distribution of the pollutant parameter.

The results are presented overall and by region. Overall, the median (25th–75th quartile) for the number of cases was 1192 (569.25–3183.25), number of deaths was 25 (8.25–54), PM2.5 was 36 (25–53), CO was 2 (1–4), O_3_ 26 (13–40), and NO_2_ 8 (3–14) Figure 2. Moreover, in Figure 3 and Figure 4, median trend levels of pollutant parameters are shown according to region and month.

Based on Spearman rho correlation calculations, overall, the number of cases significantly augmented with a rise in the levels of PM2.5 (ρ = 0.176, *p <* 0.001), CO (ρ = 0.354, *p <* 0.001), NO_2_ (ρ = 0.795, *p <* 0.001) and O_3_ (ρ = 0.057, *p <* 0.001). Furthermore, the number of deaths increased significantly with increasing concentrations of CO (ρ = 0.084, *p* = 0.001), NO_2_ (ρ = 0.156, *p <* 0.001) and O_3_ (ρ = 0.155, *p <* 0.001). The relationship between PM2.5 and deaths was not statistically significant (ρ = 0.029, *p =* 0.270).

**Figure 5:**
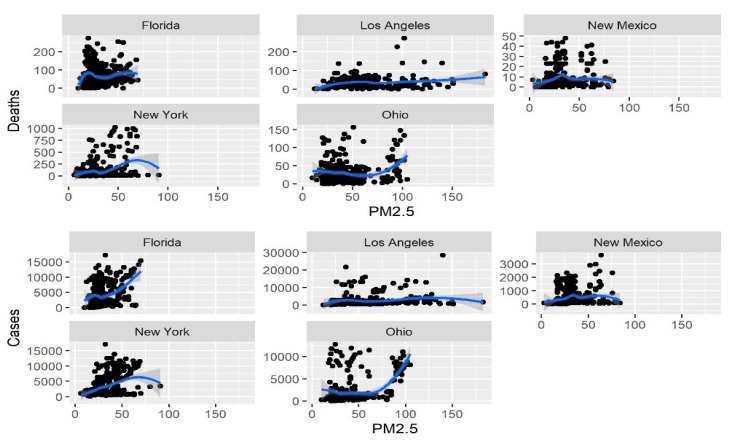
Relationship of PM2.5 concentration with the number of new daily cases and deaths.

**Figure 6:**
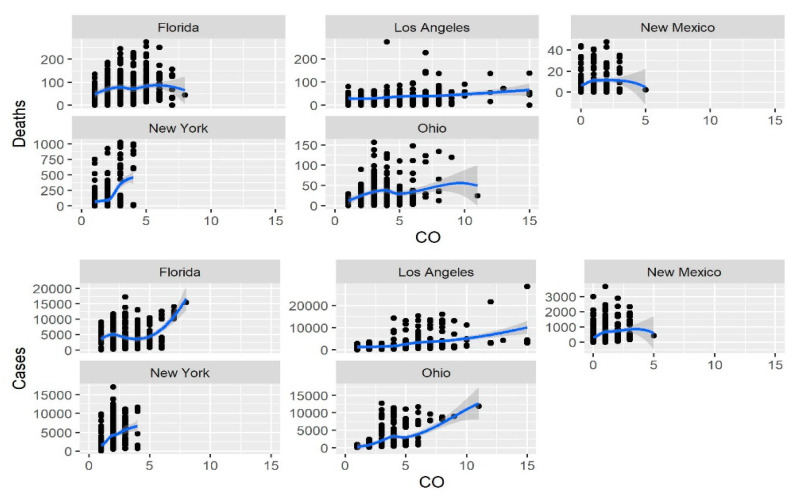
Relationship of the CO concentration with the number of new daily cases and deaths.

**Figure 7:**
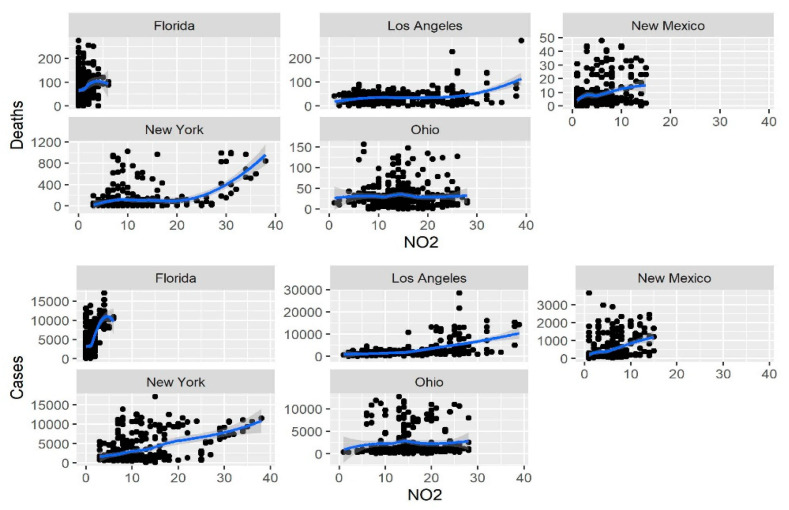
Relationship of the NO_2_ concentration with the number of new daily cases and deaths.

**Figure 8:**
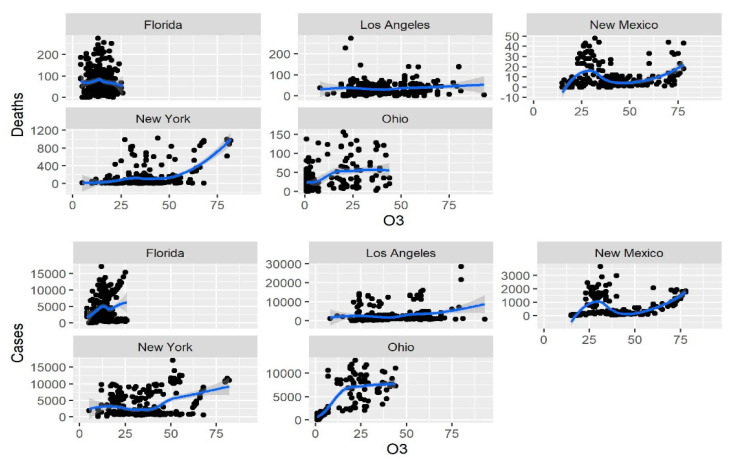
Relationship of the O_3_ concentration with the number of new daily cases and deaths.

In each of the five studied regions (Los Angeles, New Mexico, New York, Ohio, and Florida), cases and deaths displayed a significant increase with the increase in the level of the four pollutants (PM2.5, CO, NO_2_ and O_3_); results are presented in Table 1 and Table 2. These relationships in the same five USA regions between new cases and deaths for the same four pollutants are also shown graphically in Figure 5, Figure 6, Figure 7 and Figure 8.

Poisson regression results showed that, for each 1 unit increase in PM2.5, CO, NO_2_ and O_3_ concentrations, the number of cases significantly increased by 0.1%, 14.8%, 1.1%, and 0.1%, respectively. Furthermore, for each 1 unit increase in CO, NO_2_ and O_3_ concentrations, the number of deaths also significantly increased by 4.2%, 3.4%, and 1.5%, respectively. However, the regression relationship between PM2.5 and deaths was not statistically significant. These Poisson regression results are presented in Table 3 and Table 4.

## 4. Discussion

**Figure 9:**
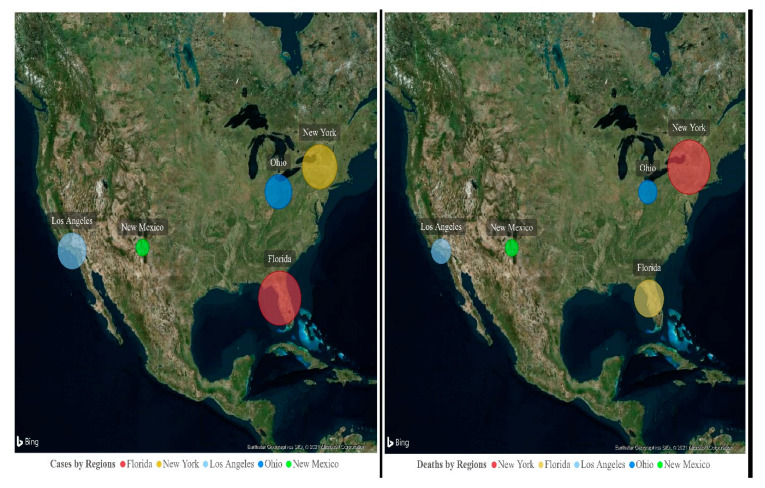
Heat map: spread of SARS-CoV-2 cases and deaths in five different regions of the USA.

**Figure 10:**
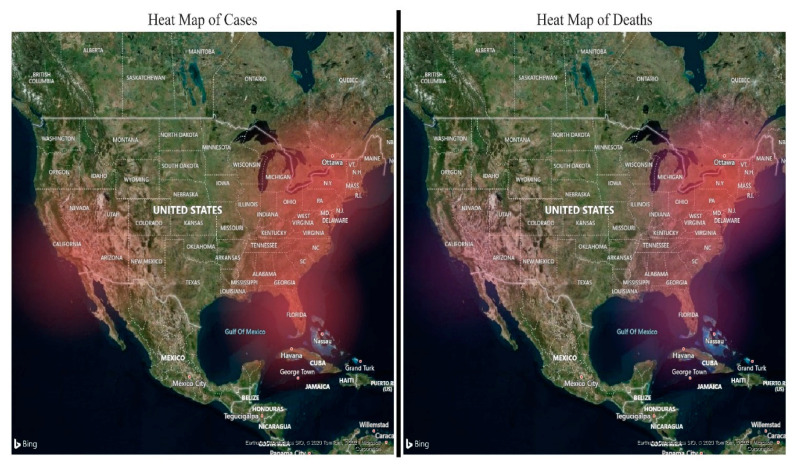
Map: the size of the bubble depicts the volume of SARS-CoV-2 cases and deaths in each region.

The present study investigated the impact of the environmental pollutants PM2.5, CO, NO_2_ and O_3_ in five USA regions and their associations with daily new cases and deaths due to the SARS-CoV-2 infection from 13 March to 31 December 2020. The study finding details the spread and the volume of SARS-CoV-2 cases and deaths in five different regions of the USA (Figure 9 and Figure 10).

Recent literature shows that local weather conditions and environmental pollution affect the regional incidence of COVID-19 [7,8,9,15,16]. It has also been reported that metropolitan areas have higher levels of environmental pollution and higher rates of SARS-CoV-2-related new daily cases and deaths. These relationships have been attributed to high particulate matter or Ozone levels [17]. In another study, Coccia (2020) [18,19] demonstrated that geographic, demographic, climatic, and environmental elements influence the spread of infectious diseases, particularly in urban areas.

Meo et al. (2020) [7], Meo et al. (2020) [8] conducted a study on the weather conditions of the Middle East and European countries and their association with SARS-CoV-2 cases and deaths in those regions. The authors found that the outbreak of SARS-CoV-2 was significantly linked to climate conditions. The lower ambient temperature was linked to a higher incidence of SARS-CoV-2. Correspondingly, in another study, Meo et al., in 2020 [9] uncovered a similar finding in Africa, where the lower ambient temperature was linked to a higher incidence of SARS-CoV-2.

The literature also established an association between environmental pollution and increased SARS-CoV-2 infections [20]. Paital and Agrawal (2020) [21] demonstrated an association between “PM2.5 levels, ambient NO_2_ concentrations, and ACE-2 expression”, respectively, with the severity of SARS-CoV-2 infections. Bianconi et al. (2020) [22] demonstrated that exposures to PM2.5 and PM-10 were linked to COVID-19 cases and deaths in Italy. Similarly, Zhu et al. (2020) [23] identified a positive linkage between “PM2.5, PM10, CO, O_3_” concentrations and the COVID-19 pandemic in China. Moreover, Frontera et al. (2020) [24] reported that rising concentrations of PM2.5 and NO_2_ caused a greater incidence of mortality from SARS-CoV-2 infections.

Bilal et al. [11] demonstrated that PM2.5, O_3_, and NO_2_ concentrations were closely related to COVID-19 outbreaks. In another study, Bashir et al. [17] found a positive association between “PM10, PM2.5, SO_2_, NO_2_, CO” and the COVID-19 pandemic in California. Moreover, Chakrabarty et al. [25] Meo et al. [26] reported that exposure to the environmental pollutant PM2.5 increased people’s susceptibility to COVID-19. In the USA, a recent study by Meo et al. [16] found that “PM2.5, CO, and O_3_” are positively associated with the number of SARS-CoV-2 daily cases and deaths. The findings of this study on the daily cases and daily deaths in five different regions of the USA are in agreement with the results demonstrated by Bilal et al. [11]; Bashir et al. [17]; Chakrabarty et al. [25]; Meo et a., [16] and Meo et al. [26].

The epidemiological and pathophysiological links are established between environmental pollution and SARS-CoV-2. Environmental pollutants act as carriers of the virus, impair immunity, and increase the susceptibility of humans to pathogens [27]. The literature highlights that PM2.5 induces adverse health effects, including oxidative stress, inflammation, and lung damage [28]. Moreover, air pollutants affect lung gene expression, lung cell composition, and cell-specific transcriptome [29]. Air pollutants enhance vulnerability through cellular damage and pathogenic burden in the lung cells and cause lung damage [30] and pneumonia [31]. The combined exposure to both pollutants and the SARS-CoV-2 pathogen causes potential interactions of two mechanisms that adversely affect the lung and consequently intensify the disease burden.

A recent study was conducted by Meo et al. [32] on environmental pollutants PM 2.5, CO, and O_3_ and their association with SARS-CoV-2 in London, UK. The authors identified that environmental pollutants “PM2.5, CO, and O_3_” positively linked with the rising number of SARS-CoV-2 daily cases and daily deaths. It was also reported that, with a 1 µm rise in PM2.5, the SARS-CoV-2 cases and deaths increased by 1.1% and 2.3%. A one-unit increase in CO level significantly increased the number of cases and deaths by 21.3% and 21.8%. It was observed that, with a one-unit increase in O3, the number of SARS-CoV-2 cases and deaths increased by 0.8% and 4.4% [32]. All this evidence favors the hypothesis that environmental pollutants greatly intensify the COVID-19 pandemic [32].

It is a recognized fact that the SARS-CoV-2 virus remains viable in aerosols for several hours, and this enables the rapid spread of the virus over a vast distance [33,34,35]. The viral contaminated aerosols can be inhaled deep into the lung and cause infection. PM could play a significant role in the spread of SARS-CoV- 2. Setti et al. [36] demonstrate that SARS-CoV-2 was present in PM samples obtained during the Italian COVID-19 epidemic. The findings show that PM can act as a carrier with aerosols and transmit and increase the spread of the associated virus.

In the present study, we found a piece of evidence that supports the hypothesis regarding the causal linkage between particulate matter PM2.5, CO, NO_2_, and O_3_ and the epidemiological facts of COVID-19 cases and deaths. Moreover, PM2.5, CO, NO_2_, and O_3_ are highly toxic, such that exposure to these substances can damage the lungs. Due to environmental pollutants, these mechanisms of lung injury support the hypothesis that exposure to PM2.5, CO, NO_2_, and O_3_ has increased the number of new SARS-CoV-2 cases and deaths in the five different regions in the USA.

## 5. Study Strengths and Limitations

This study investigated the effect of the environmental pollutants PM2.5, CO, NO_2_, and O_3_ on the incidence and mortality trends of SARS-CoV-2 infection in five different regions in the USA. We selected PM 2.5, CO, NO_2_, and O_3_ as these pollutants deeply penetrating the lungs. Moreover, we decided on these five USA regions because they are affected by many environmental contaminants and SARS-CoV-2. The daily new cases, daily deaths, and concentrations of PM2.5, CO, NO_2_, and O_3_ were recorded for an extended period from 13 March to 31 December 2020. Limited studies have been conducted to investigate the effects of environmental pollutants PM2.5, CO, NO_2_, and O_3_ on SARS-CoV-2 daily cases and death for as long as ten months. The sampling and nature of this study cannot necessarily identify relevant other factors that might affect the relationship between exposure to environmental pollutants and the development or severity of COVID-19 infections. COVID-19 cases and deaths may increase for different reasons in addition to pollution, such as patient characteristics, genetics, co-morbidities, and social, economic, and community-level risk factors. Another constraint of this study is that we could not obtain the data for other air pollutants, such as PM 10, carbon dioxide, sulphur dioxide, and temperature and humidity, which may also affect the occurrence and mortality of SARS-CoV-2.

## 6. Conclusions

Empirical data from this study demonstrate that PM2.5, CO, NO_2_, and O_3_ are associated with SARS-CoV-2 daily cases and deaths in five different regions of the USA. These findings can help inform health officials and policymakers to combat the COVID-19 pandemic outbreak in these USA regions and stress the necessity of reducing the environmental pollution. The conclusions of this study highlight the critical contributions of PM2.5, CO, NO_2_, and O_3_ as triggering factors for new cases and mortality due to the COVID-19 pandemic. The findings here favor a hypothesis that exposure to environmental pollutants can cause more lethal forms of COVID-19.

## Figures and Tables

**Table 1:** Correlation of PM2.5, CO, NO_2_ and O_3_ with the number of SARS-CoV-2 cases by region.

Cases by Region	Environmental Pollutants	Spearman’s ρ	*p*-Value
Florida	Particulate matter (PM2.5)	0.355	<0.001 *
	Carbon monoxide (CO)	0.182	<0.001 *
	Nitrogen dioxide (NO_2_)	0.597	<0.001 *
	Ozone (O_3_)	0.198	0.001 *
Los Angeles	Particulate matter (PM2.5)	0.207	<0.001 *
	Carbon monoxide (CO)	0.394	<0.001 *
	Nitrogen dioxide (NO_2_)	0.544	<0.001 *
	Ozone (O_3_)	0.202	0.001 *
New Mexico	Particulate matter (PM2.5)	0.132	0.024 *
	Carbon monoxide (CO)	0.326	<0.001 *
	Nitrogen dioxide (NO_2_)	0.384	<0.001 *
	Ozone (O_3_)	0.173	0.003 *
New York	Particulate matter (PM2.5)	0.432	<0.001 *
	Carbon monoxide (CO)	0.448	<0.001 *
	Nitrogen dioxide (NO_2_)	0.468	<0.001 *
	Ozone (O_3_)	0.347	<0.001 *
Ohio	Particulate matter (PM2.5)	0.394	<0.001 *
	Carbon monoxide (CO)	0.468	<0.001 *
	Nitrogen dioxide (NO_2_)	0.035	<0.001 *
	Ozone (O_3_)	0.820	<0.001 *

* Statistically significant at 5% level of significance.

**Table 2:** Correlation of PM2.5, CO, NO_2_ and O_3_ with the number of deaths due to SARS-CoV-2.

Deaths by Region	Environmental Pollutants	Spearman’s ρ	*p*-Value
Florida	Particulate matter (PM2.5)	0.04	0.948
	Carbon monoxide (CO)	0.170	0.003 *
	Nitrogen dioxide (NO_2_)	0.207	<0.001 *
	Ozone (O_3_)	0.021	0.726
Los Angeles	Particulate matter (PM2.5)	0.141	0.017 *
	Carbon monoxide (CO)	0.213	0.017 *
	Nitrogen dioxide (NO_2_)	0.307	<0.001 *
	Ozone (O_3_)	0.076	0.196
New Mexico	Particulate matter (PM2.5)	0.052	0.376
	Carbon monoxide (CO)	0.190	<0.001 *
	Nitrogen dioxide (NO_2_)	0.288	<0.001 *
	Ozone (O_3_)	0.066	0.256
New York	Particulate matter (PM2.5)	0.328	<0.001 *
	Carbon monoxide (CO)	0.431	<0.001 *
	Nitrogen dioxide (NO_2_)	0.468	<0.001 *
	Ozone (O_3_)	0.476	<0.001 *
Ohio	Particulate matter (PM2.5)	0.146	0.013 *
	Carbon monoxide (CO)	0.202	0.001 *
	Nitrogen dioxide (NO_2_)	0.012	0.841
	Ozone (O_3_)	0.407	<0.001 *

* Statistically significant at 5% level of significance.

**Table 3:** Poisson Regression—PM2.5, CO, NO_2_ and O_3_ with the number of cases due to SARS-CoV-2.

Environmental Pollutants	B	S.E	Exp (β)	*p*-Value
Particulate matter (PM2.5)	0.001	0.000231	1.001	<0.001 *
Carbon monoxide (CO)	0.138	0.0002	1.148	<0.001 *
Nitrogen dioxide (NO_2_)	0.011	0.000653	1.011	<0.001 *
Ozone (O_3_)	0.001	0.000269	1.001	<0.001 *

* Statistically significant at 5% level of significance: S.E = standard error. β = coefficient estimates; Exp (β) = exponentiated values; Wald = explanatory variables.

**Table 4:** Poisson Regression—PM2.5, CO, NO_2_ and O_3_ with the number of deaths due to SARS-CoV-2.

Environmental Pollutants	B	S.E	Exp (β)	*p*-Value
Particulate matter (PM2.5)	0.008	0.0002	0.993	0.578
Carbon monoxide (CO)	0.041	0.0015	1.042	<0.001 *
Nitrogen dioxide (NO_2_)	0.034	0.0004	1.034	<0.001 *
Ozone (O_3_)	0.015	0.0002	1.015	<0.001 *

* Statistically significant at 5% level of significance: S.E = standard error. β = coefficient estimates; Exp (β) = exponentiated values; Wald = explanatory variables.

## Data Availability

Data may be provided on reasonable request to corresponding author.
